# Molecular Signatures for the PVC Clade (Planctomycetes, Verrucomicrobia, Chlamydiae, and Lentisphaerae) of Bacteria Provide Insights into Their Evolutionary Relationships

**DOI:** 10.3389/fmicb.2012.00327

**Published:** 2012-09-17

**Authors:** Radhey S. Gupta, Vaibhav Bhandari, Hafiz Sohail Naushad

**Affiliations:** ^1^Department of Biochemistry and Biomedical Sciences, McMaster UniversityHamilton, ON, Canada

**Keywords:** conserved signature indels, signature proteins, Verrucomicrobia, Planctomycetes, Chlamydia, Lentisphaerae, PVC superphylum, phylogenetic trees

## Abstract

The PVC superphylum is an amalgamation of species from the phyla Planctomycetes, Verrucomicrobia, and Chlamydiae, along with the Lentisphaerae, Poribacteria, and two other candidate divisions. The diverse species of this superphylum lack any significant marker that differentiates them from other bacteria. Recently, genome sequences for 37 species covering all of the main PVC groups of bacteria have become available. We have used these sequences to construct a phylogenetic tree based upon concatenated sequences for 16 proteins and identify molecular signatures in protein sequences that are specific for the species from these phyla or those providing molecular links among them. Of the useful molecular markers identified in the present work, six conserved signature indels (CSIs) in the proteins Cyt c oxidase, UvrD helicase, urease, and a helicase-domain containing protein are specific for the species from the Verrucomicrobia phylum; three other CSIs in an ABC transporter protein, cobyrinic acid ac-diamide synthase, and SpoVG protein are specific for the Planctomycetes species. Additionally, a 3 aa insert in the RpoB protein is uniquely present in all sequenced Chlamydiae, Verrucomicrobia, and Lentisphaerae species, providing evidence for the shared ancestry of the species from these three phyla. Lastly, we have also identified a conserved protein of unknown function that is exclusively found in all sequenced species from the phyla Chlamydiae, Verrucomicrobia, Lentisphaerae, and Planctomycetes suggesting a specific linkage among them. The absence of this protein in Poribacteria, which branches separately from other members of the PVC clade, indicates that it is not specifically related to the PVC clade of bacteria. The molecular markers described here in addition to clarifying the evolutionary relationships among the PVC clade of bacteria also provide novel tools for their identification and for genetic and biochemical studies on these organisms.

## Introduction

The bacteria of the Planctomycetes, Verrucomicrobia, Chlamydiae, and Lentisphaerae phyla along with the Candidate Poribacteria, Candidate phylum OP3 and Candidate division WWE2 are collectively grouped and referred to as the PVC superphylum or the PVC clade (Wagner and Horn, 2006). The PVC group is comprised of species that are of much importance due to their characteristics and the roles they play in many areas of life. Species of the Chlamydiae phylum are one of the most widely studied microorganisms due to their pathogenic capacities in humans and in animals. They are responsible for many human illnesses including sexually transmitted urinary tract infections, trachoma, and pneumonia (Sachse et al., 2009). Species of the phylum Planctomycetes are renowned for their unusual cellular features such as internal compartmentalization, sterol biosynthesis, and endocytosis-analogous pathways that are generally associated with the eukaryotes (Fuerst and Webb, 1991; Lindsay et al., 1997; Pearson et al., 2003; Ward et al., 2006; Lonhienne et al., 2010; Fuerst and Sagulenko, 2011; McInerney et al., 2011). This phylum also harbors a group of anaerobic chemoautotrophic “anammox” (anaerobic ammonium oxidation) organisms (van de Graaf et al., 1995; Strous et al., 1999). These anammox species can oxidize ammonium to dinitrogen and are therefore quite useful in decontamination of wastewater rich in ammonia (Dalsgaard et al., 2003). Their importance is underscored by estimates which suggest that anammox bacteria may contribute up to 50% of the atmospheric nitrogen (Devol, 2003). The species from the phylum Verrucomicrobia are abundant in soil based environments with estimates proposing that up to 10% of all bacteria in the soil belong to this phylum (Sangwan et al., 2005). These bacteria are also found in aquatic environments (Martiny et al., 2005; Haukka et al., 2006) and known to associate with eukaryotic species as indicated by their presence in termite guts, human intestines, nematodes, and some ciliate protozoa (Petroni et al., 2000; Vandekerckhove et al., 2002; Shinzato et al., 2005; Wang et al., 2005). Some members of the Verrucomicrobiae are known to exist in ultramicrobial sizes, others to possess extensions of the cellular membrane termed the prosthecae and some also exist in acidophilic environments (Hedlund et al., 1997; Janssen et al., 1997; Pol et al., 2007). Thus, the species of the PVC phylum are important in our quest to better understand prokaryotic evolution, microbial ecology, and physiology.

Though much diversity exists among the bacteria of different phyla that comprises this superphylum, a close relationship among them has been suggested by the 16S rRNA trees and number of other phylogenetic studies employing single gene and multi-gene analyses of protein sequences (Cho et al., 2004; Wagner and Horn, 2006; Hou et al., 2008; Pilhofer et al., 2008; Glockner et al., 2010; Siegl et al., 2011). Among the members of this clade, the Planctomycetes and Chlamydiae were observed to be phylogenetically related as early as 1986 based on 16S rRNA secondary structures and phylogenetic trees (Weisburg et al., 1986; Woese, 1987; Fuerst, 1995). A close relationship of the Verrucomicrobia to the Chlamydiae and Planctomycetes was first observed by Hedlund et al. (1996) and the “sister-taxon” grouping of the Lentisphaerae to the Verrucomicrobia was recognized with the isolation of the first Lentisphaerae organism *Victivallis vadensis* (Zoetendal et al., 2003; Cho et al., 2004). The taxonomic entity labeled as the PVC superphylum was proposed in 2006, based on 16S ribosomal data, by Wagner and Horn (2006) to encompass the monophyletic group comprised of the above four phyla along with the recently discovered Candidate Poribacteria, Candidate phylum OP3 and Candidate phylum WWE2 (Hugenholtz et al., 1998; Fieseler et al., 2004; Chouari et al., 2005; Wagner and Horn, 2006). However, a monophyletic grouping of the different bacteria belonging to these phyla has also been disputed by other phylogenetic studies based upon 16S rRNA as well as several single gene and concatenated protein phylogenies (Ward et al., 2000; Jenkins and Fuerst, 2001; Ciccarelli et al., 2006; Griffiths and Gupta, 2007; Santarella-Mellwig et al., 2010).

Apart from their linkages in phylogenetic trees, little evidence exists to group the different phyla that are part of the PVC clade into a single large group. Nevertheless, some uncommon features are seen to be shared by multiple phyla of the group. The Verrucomicrobia along with the Poribacteria and Lentisphaerae share a similar intracellular structural plan with the Planctomycetes in having membranous borders dividing the cell into compartments (Fieseler et al., 2004; Lee et al., 2009; Fuerst and Sagulenko, 2011). Planctomycetes and Chlamydiae lack peptidoglycan in their cell walls (Konig et al., 1984; Liesack et al., 1986; Fox et al., 1990; Staley et al., 1992; Ward et al., 2006; Fuerst and Sagulenko, 2011). Also common among the Chlamydiae and Planctomycetes is the lack of FtsZ-based cell division (Bernander and Ettema, 2010; Fuerst and Sagulenko, 2011). However, as these features are not exclusive to the members of the PVC group and not found in all species of the phyla comprising the PVC group, they do not provide much clarity in the debate concerning the grouping of these phyla into a superphylum.

Due to the advent of rapid genomic sequencing techniques and availability of genomic sequences, comparative genomics provide powerful means for answering a variety of questions related to bacterial evolution. Using genome sequences, many approaches are being used to understand the evolutionary relationships among bacteria. While some approaches using whole genome alignments have been most used (or are mainly applicable) for studying closely related organisms (Angiuoli and Salzberg, 2011; Agren et al., 2012; Sahl et al., 2012), other comparative genomic approaches involving identification of molecular markers in the forms of either conserved signature inserts or deletions (CSIs) or conserved signature proteins (CSPs) have been extensively used to define taxonomic clades of different phylogenetic ranks in molecular terms (Gupta, 1998, 2010; Gupta and Griffiths, 2002; Dutilh et al., 2008; Gao and Gupta, 2012). The applications of these approaches previously to the Chlamydiae species have led to identification of numerous CSIs and CSPs that are specific for the species from this phylum or a number of its subclades (Griffiths et al., 2005, 2006; Gupta and Griffiths, 2006). Some interesting cases of lateral gene transfers (LGTs) between Actinobacteria and Chlamydiae were also identified by these studies (Griffiths and Gupta, 2006). Additionally, our work using these approaches also indicated that the phyla Chlamydiae and Verrucomicrobia are specifically related and they shared a common ancestor exclusive of the Planctomycetes (Griffiths and Gupta, 2007). However, thus far no molecular markers have been identified that are specific for the Planctomycetes and/or Verrucomicrobia phyla or those linking all members of the PVC group. In the present work, we describe the results of comparative genomic analysis aimed at identifying molecular markers that are uniquely shared by either the Planctomycetes or Verrucomicrobia phyla or those that are commonly shared by different main groups of the PVC superphylum. Additionally, we also report phylogenetic studies based upon concatenated protein sequences to evaluate the relationships among the PVC clade of bacteria.

## Materials and Methods

Complete or partial genomic sequences are now available for 37 species/strains belonging to the PVC group (see Table 1). For phylogenetic analyses, sequences for 16 housekeeping and ribosomal proteins (ArgRS, EF-G, EF-Tu, GyrA, GyrB, DnaK, IleRS, RecA, RpoB, RpoC, TrpRS, UvrD, ValRS along with ribosomal proteins L1, L5, and S12) were utilized. The protein sequences for various species of the PVC group and for species from some other bacterial phyla were retrieved from the NCBI protein database and their alignments were constructed using the ClustalX 1.83 program (Jeanmougin et al., 1998; NCBI protein database, 2012). After concatenation of all of these sequence alignments into a single file, the poorly aligned regions were removed using the Gblocks_0.91b program (Castresana, 2000). The remaining 7016 aligned and homologous characters were employed for construction of phylogenetic trees using the neighbor-joining (NJ) and maximum likelihood (ML) algorithms as described in our earlier work (Gupta and Mok, 2007; Gupta and Bhandari, 2011; Naushad and Gupta, 2012).

**Table 1:** **Some characteristics for sequenced species of the PVC group of bacteria**.

Organism	GC%	Size (Mb)	Ref seq identity	Genome status	No. of proteins	Reference
**PLANCTOMYCETES**						
*Candidatus Kuenenia stuttgartiensis*	41.0	4.2	–	Draft	4663	Strous et al. (2006)
*Phycisphaera mikurensis*	73.0	3.9	NC_017080.1	Complete	3287	NCBI genome project
*Gemmata obscuriglobus*	67.2	9.2	NZ_ABGO00000000	Draft	7989	JCVI
*Isosphaera pallida*	62.4	5.5	NC_014962.1	Complete	3722	Goker et al. (2011)
*Singulisphaera acidiphila*	59.9	9.7	NZ_AGRX00000000	Draft	7630	DOE-JGI*
*Rhodopirellula baltica*	55.4	7.1	NC_005027.1	Complete	7325	Glockner et al. (2003)
*Pirellula staleyi*	57.5	6.2	NC_013720.1	Complete	4717	Clum et al. (2009)
*Blastopirellula marina*	57.0	6.6	NZ_AANZ00000000	Draft	6025	Glockner et al. (2003)
*Planctomyces limnophilus*	53.7	5.5	NC_014148.1	Complete	4258	Labutti et al. (2010)
*Planctomyces brasiliensis*	56.4	6.0	NC_015174.1	Complete	4750	DOE-JGI*
*Planctomyces maris*	50.5	7.8	NZ_ABCE00000000	Draft	6480	JCVI
**VERRUCOMICROBIA**						
*Opitutaceae bacterium Tav5*	61.0	7.4	NZ_AGJF00000000	Draft	6006	DOE-JGI*
*Opitutaceae bacterium Tav1*	63.2	7.1	NZ_AHKS00000000	Draft	5984	DOE-JGI*
*Diplosphaera colitermitum*	60.7	5.2	NZ_ABEA00000000	Draft	4826	DOE-JGI*
*Opitutus terrae*	55.3	6.0	NC_010571.1	Complete	4612	van Passel et al. (2011a)
*Coraliomargarita akajimensis*	53.6	3.7	NC_014008.1	Complete	3120	Mavromatis et al. (2010)
*Verrucomicrobiae bacterium DG1235*	54.3	5.8	NZ_ABSI00000000	Draft	4909	JCVI
*Methylacidiphilum infernorum*	45.5	2.3	NC_010794.1	Complete	2472	Hou et al. (2008)
*Pedosphaera parvula*	52.6	7.4	NZ_ABOX00000000	Draft	6510	Kant et al. (2011b)
*Akkermansia muciniphila*	55.8	2.7	NC_010655.1	Complete	2138	DOE-JGI*
*Verrucomicrobium spinosum*	60.3	8.2	NZ_ABIZ00000000.1	Complete	6509	TIGR^#^
*Chthoniobacter flavus*	61.1	7.8	NZ_ABVL00000000	Draft	6716	Kant et al. (2011a)
**CHLAMYDIAE**						
*Chlamydophila abortus*	39.9	1.1	NC_004552.2	Complete	932	Thomson et al. (2005)
*Chlamydophila psittaci*	39.1	1.2	NC_017289.1	Complete	975	Schofl et al. (2011)
*Chlamydophila caviae*	39.1	1.2	NC_003361.3	Complete	1005	Read et al. (2003)
*Chlamydophila felis*	39.3	1.2	NC_007899.1	Complete	1054	Azuma et al. (2006)
*Chlamydophila pecorum*	41.1	1.1	NC_015408.1	Complete	988	Mojica et al. (2011)
*Chlamydophila pneumoniae*	40.6	1.2	NC_002179.2	Complete	1119	Read et al. (2000)
*Chlamydia trachomatis*	41.3	1.0	NC_010287.1	Complete	874	Thomson et al. (2008)
*Chlamydia muridarum*	40.3	1.1	NC_002620.2	Complete	910	Read et al. (2000)
*Simkania negevensis*	41.6	2.6	NC_015713.1	Complete	2518	Collingro et al. (2011)
*Waddlia chondrophila*	43.8	2.1	NC_014225.1	Complete	1956	Bertelli et al. (2010)
*Parachlamydia acanthamoebae*	39.0	3.1	NC_015702.1	Complete	2789	Collingro et al. (2011)
*Protochlamydia amoebophila*	34.7	2.4	NC_005861.1	Complete	2031	Horn et al. (2004)
**LENTISPHAERAE AND PORIBACTERIA**						
*Victivallis vadensis* Lentisphaerae	59.4	5.3	NZ_ABDE00000000	Draft	4065	van Passel et al. (2011b)
*Lentisphaera araneosa*	41.0	6.0	NZ_ABCK00000000	Draft	5104	Thrash et al. (2010)
*Candidatus Poribacteria WGA-A3*	53.4	1.9	NZ_ADFK00000000	Draft	1585	Siegl et al. (2011)

*DOE-JGI – U.S. Department of Energy Joint Genomic Institute.

*^#^TIGR – The Institute for Genomic Research*.

*JCVI – J. Craig Venter Institute*.

Identification of CSIs that are specific for the PVC group of species was carried out using similar procedures as described in our earlier work (Griffiths et al., 2005; Gupta and Bhandari, 2011; Naushad and Gupta, 2012). Briefly, BlastP searches were initially conducted on various proteins from the genomes of *Opitutus terrae* (van Passel et al., 2011a) and *Pirellula staleyi* (Clum et al., 2009) and sequences for 10–12 species that included assorted species from the PVC group and some from other phyla were retrieved. Sequence alignments for these proteins were created and manually examined for inserts or deletions that were flanked on both sides by conserved regions (Gupta and Griffiths, 2002; Gupta and Bhandari, 2011; Naushad and Gupta, 2012). A second, more detailed BlastP search was then carried out on the identified sequence consisting of the indel and the conserved flanking region. The indels that were specific for the members of the PVC group were formatted into signature files showing the sequence alignments and GenBank identifier (GI) numbers of various proteins.

## Results

### Phylogenetic analyses of the PVC group of bacteria based upon concatenated protein sequences

**Figure 1:**
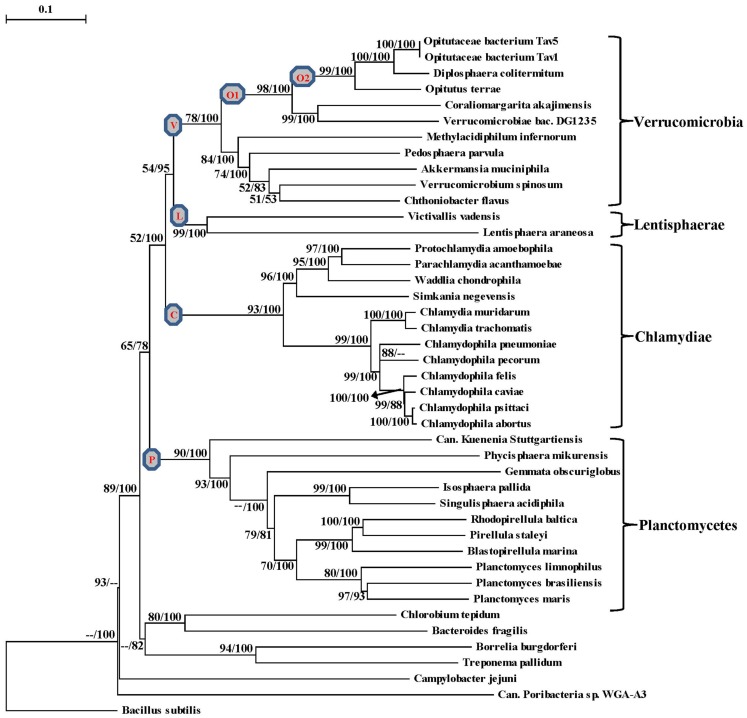
**A Neighbor-joining distance tree for the sequenced species belonging to the PVC group of bacteria based upon concatenated sequences for 16 conserved proteins**. The numbers on the node indicate% statistical support for different nodes in the ML and NJ analyses, respectively. The scores that were less than 50% are not shown and represented by (–). The letters in the circle mark separate clades for the Verrucomicrobia phylum (V), Planctomycetes phylum (P), Chlamydiae (C), Lentisphaerae (L), Opitutae class (O1), Opitutaceae family (O2).

The proposal to amalgamate different bacterial groups that are part of the PVC clade is mainly based upon their branching in the 16S rRNA trees (Wagner and Horn, 2006). As indicated earlier, although close branching of species from some of these groups has been observed in a number of studies (Cho et al., 2004; Wagner and Horn, 2006; Hou et al., 2008; Pilhofer et al., 2008; Glockner et al., 2010; Siegl et al., 2011) most of these studies did not contain representatives from all bacterial phyla that are part of the PVC clade and their results have been contradicted by other analyses (Ward et al., 2000; Ciccarelli et al., 2006; Griffiths and Gupta, 2007). It is now widely accepted that in contrast to phylogenetic inferences based upon any single gene or protein, including 16S rRNA, those based upon large numbers of characters derived from multiple conserved genes/proteins are more reliable in accurately depicting the evolutionary relationships among distantly related phyla (Rokas et al., 2003; Ciccarelli et al., 2006; Wu and Eisen, 2008). Although some earlier studies are based upon concatenated protein sequences, they contained only limited numbers of Chlamydiae or Planctomycetes species (generally 4–5 Chlamydiaceae and 1–2 Planctomycetes) and no representative from the Verrucomicrobia or Lentisphaerae phyla (Ciccarelli et al., 2006; Strous et al., 2006; Hou et al., 2008). Our earlier work based upon concatenated protein sequences also included only one Verrucomicrobiae and three Planctomycetes species (Griffiths and Gupta, 2007). However, complete or partial genomic sequences are now available for 37 species belonging to the PVC clade of bacteria, including 11 species each from the Planctomycetes and Verrucomicrobia phyla, 12 from the Chlamydiae, two from the Lentisphaerae and a Poribacteria (Table 1). Hence, to examine the evolutionary relationship among these species, phylogenetic trees were constructed based upon a large concatenated dataset of protein sequences derived from 16 important proteins (see Methods). Most of these proteins are universally distributed and have been extensively used for phylogenetic analyses (Ciccarelli et al., 2006; Strous et al., 2006; Gupta and Mok, 2007; Hou et al., 2008). The trees were constructed using both ML and NJ methods and the results of these studies are summarized in Figure 1. The numbers at the nodes in this tree show the statistical significance of the node by the ML and NJ methods, respectively.

In the tree based upon concatenated protein sequences (Figure 1), species of the Planctomycetes, Verrucomicrobia, Chlamydiae, and Lentisphaerae phyla branched together with other members of their phylum. The monophyly and distinctness of these clades was well supported by both ML and NJ analyses with at least 75% bootstrap support by each of these methods. In this tree, Lentisphaerae and Verrucomicrobia were observed to branch together. Although a clade consisting of these two phyla has a bootstrap score of 95% by the NJ method, it was very weakly supported (supported only 54% of the time) by the ML method. Similarly, a clade consisting of the Lentisphaerae, Verrucomicrobia and Chlamydiae phyla was also strongly supported by the NJ method but not by the ML analysis. Additionally, although in this tree the four phyla that form the PVC clade were observed to branch together, a clade consisting of all four of them was poorly supported by both ML and NJ methods. Lastly, the single Poribacteria species in our dataset did not branch with the PVC group of bacteria. In addition to these observations, this tree also provides some insights into the relationships within the Verrucomicrobia and Planctomycetes phyla, which are discussed below together with the results of signature sequences for these groups of bacteria.

### Phylogeny and molecular signatures for the phylum verrucomicrobia

The sequenced Verrucomicrobia species formed a distinct clade in our phylogenetic tree (Figure 1), which was strongly supported by the NJ method and also had significant support by the ML analysis. Within this clade, the different Verrucomicrobia species split into two main clades, both of which were significantly supported by the NJ and ML analyses. One of these clades (marked **O1**), which we will refer to as the Opitutae clade, was comprised of the species *O. terrae*, *Diplosphaera colitermitum, Coraliomargarita akajimensis, Opitutaceae bacterium TAV5*, and *TAV1* and also *Verrucomicrobiae bacterium DG1235*. The first five of these species/strains belong to the class Opitutae, whereas *V. bacterium DG1235* is currently a part of the class Verrucomicrobiae (NCBI Taxonomy, 2012). The other members of the class Verrucomicrobiae (viz. *Verrucomicrobium spinosum*, *Akkermansia muciniphila* and *Pedosphaera parvulaparvula*) were part of the second major clade where they branched with *Chthoniobacter flavus*, a member of the class Spartobacteria and *Methylacidiphilum infernorum*, an unclassified species belonging to this phylum (Yoon et al., 2008; NCBI Taxonomy, 2012).

**Figure 2:**
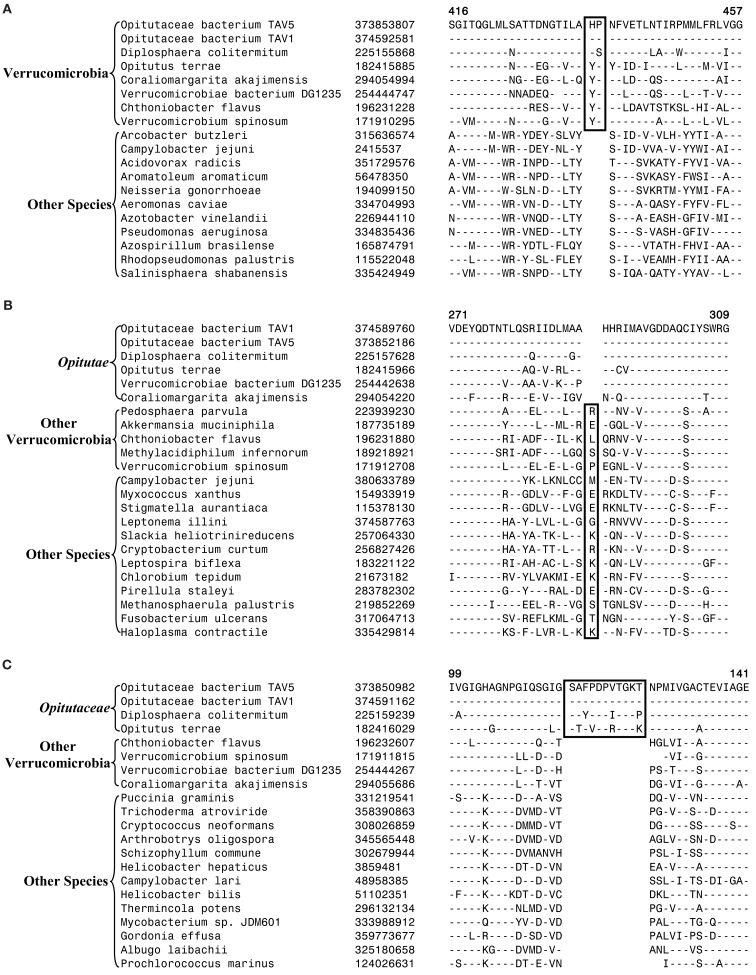
**Partial sequence alignments of three different proteins showing CSIs that are specific for the Verrucomicrobia species**. **(A)** A 2 aa CSI in a conserved region of Cytochrome c oxidase (cbb3-type) subunit 1 that is specific for all sequenced Verrucomicrobia species where homologs of this protein were identified; **(B)** A CSI consisting of 1 aa deletion in the UvrD helicase that is specific for the Opitutae class; and **(C)** An 11 aa insert in the Urease alpha subunit that is specific for the Opitutaceae family. The CSIs are boxed and the dashes (–) in this and all other alignments indicate identity with the amino acid that is present on the top line. The position of these sequence regions for the species on the top line is noted above the sequence. Except for the indicated groups of Verrucomicrobia, these CSIs are not present in any other species in the top 250 Blastp hits. Sequence information for only limited number of species from other phyla of bacteria are shown in the alignments. The GenBank identifier (GI) numbers for different proteins are shown in the second columns.

Currently, no molecular or biochemical marker of any kind is known that is specific for the species from the phylum Verrucomicrobia. However, of the signatures that we have identified, one consisting of a 2 aa insert in the Cytochrome c oxidase protein (Figure 2A) provides a potential molecular marker for this phylum. This indel is present in all members of the Verrucomicrobia phylum where the homologs of this protein could be detected, but it was not found in the homologs of this protein from any other bacteria including those from the Lentisphaerae, Chlamydiae, and Planctomycetes phyla. As this insert (CSI) is of fixed length, and it is present within a conserved region of the protein, it provides a useful and reliable molecular marker. Due to the highly specific nature of the genetic change which gave rise to this CSI and its specific presence only in this group of species, the genetic event responsible for this most likely occurred in a common ancestor of this phylum followed by vertical transmission of the gene containing this CSI to various descendant species (Gupta, 1998; Gupta and Griffiths, 2002; Gupta and Bhandari, 2011). Although a homolog for this protein was not detected in all sequenced verrucomicrobiae species, the noted genetic characteristic is specific for the species from this phylum and it provides a molecular means to distinguish species possessing the homolog from other bacteria.

**Figure 3:**
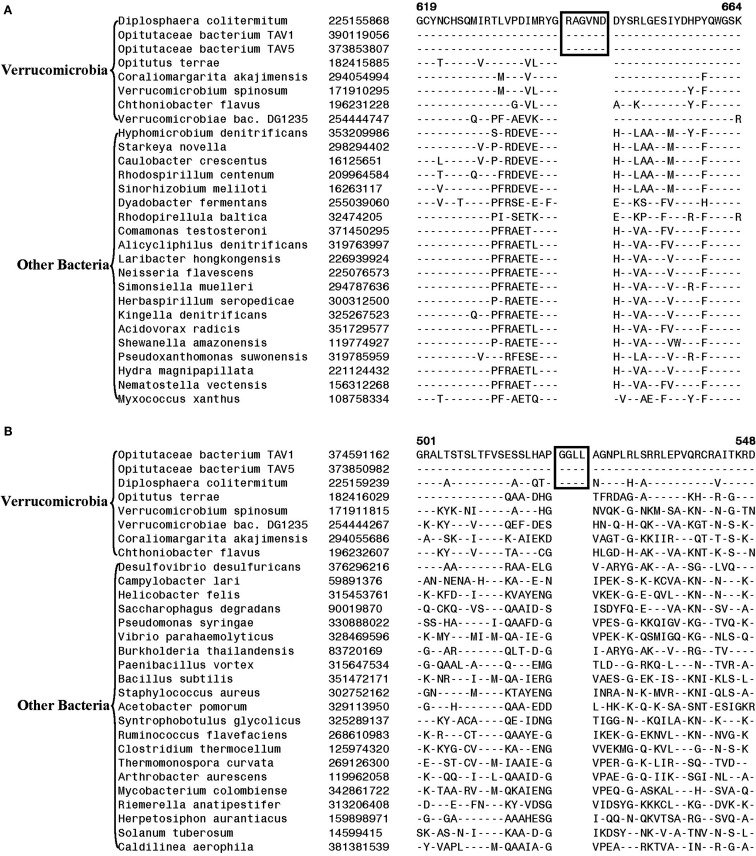
**Partial sequence alignment of (A) Cytochrome c oxidase and (B) alpha subunit of urease, showing two CSIs (boxed) that are specifically present in *D. colitermitum*, *Opitutaceae bacterium TAV1*, and *Opitutaceae bacteriumTAV5* species**.

**Figure A1:**
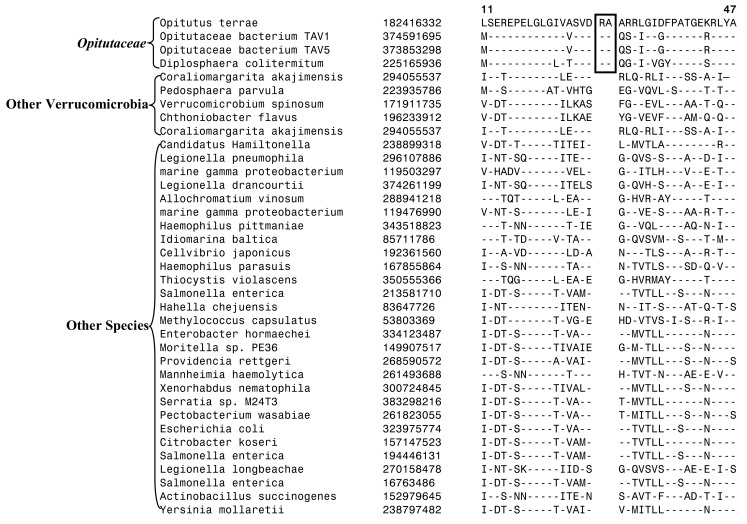
**Partial sequence alignment of the helicase domain-containing protein showing 2 aa insert that is specific for the family Opitutaceae**. The insert in not present in other Verrucomicrobia or in any other group of bacteria.

**Figure A2:**
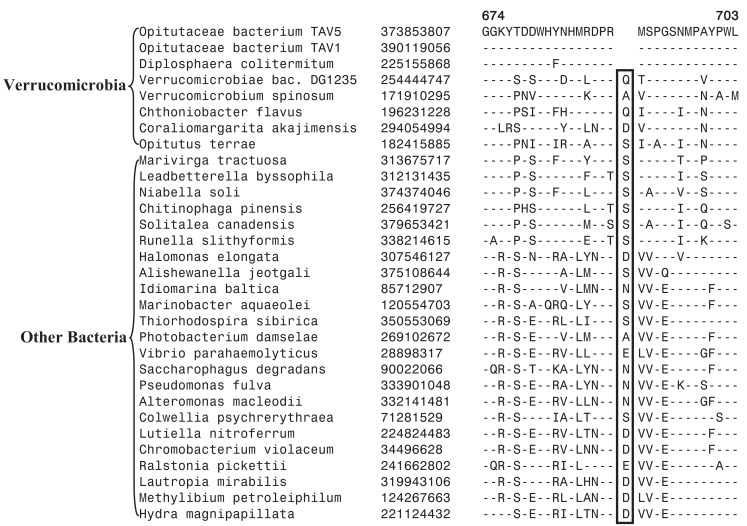
**A 1 aa deletion in a conserved region in the Cytochrome c oxidase protein is shown in this partial sequence alignment with the deletion specific for *Opitutaceae bacterium Tav1*, *Opitutaceae bacterium Tav5*, and *Diplosphaera colitermitum* species**. The three species harboring the indel also branch together in the concatenated protein tree.

Another identified CSI, shown in Figure 2B, consists of a 1 aa deletion in a conserved region of the UvrD helicase enzyme that is specific for the Opitutae clade (01) of Verrucomicrobia species (Figure 1). The species distribution of this CSI is consistent with the phylogenetic tree and it supports the grouping/placement of *V. bacterium* DG1235 within the Opitutae class rather than with other members of the class Verrucomicrobiae. The branching of *V. bacterium DG1235* with the Opitutae class of bacteria has also been observed in earlier studies (Pilhofer et al., 2008; Wertz et al., 2012). This CSI provides a potentially useful molecular marker for the Opitutae class. Within the Opitutae class, a subclade consisting of *O. terrae*, *D. colitermitum*, and *O. bacterium TAV5* and *TAV1*, which represent the Opitutaceae family of species, was also strongly supported. During our analyses, two CSIs that are specific for this subclade were identified. The sequence information for one of these CSIs consisting of an 11 aa insert in the Urease enzyme, is shown in Figure 2C. Another CSI consisting of a 2 aa insert showing similar specificity is present in a helicase domain-containing protein and sequence information for this is presented in Figure A1 in Appendix. Within the Opitutaceae family, the two unclassified species *O. bacterium TAV5* and *TAV1* exhibit closer relationship in the phylogenetic tree to *D. colitermitum* than to *O. terrae* (Yoon et al., 2008). A close relationship between these species was supported by three CSIs that were identified in the present work. The sequence information for two of these CSIs, which are present in the Cyt c oxidase and the Urease proteins are shown in Figure 3. The sequence information for another CSI (a 1 aa deletion) in the Cyt c oxidase protein that is also specific for these species is presented in Figure A2 in Appendix. It is noteworthy that these two proteins (viz. Cyt c oxidase and Urease) also contain other CSIs in different positions that are specific for the phylum Verrucomicrobia or the class Opitutae (Figures 2A,B), indicating that distinct genetic changes within these genes have occurred at different evolutionary stages.

### Phylogeny and molecular signatures for the planctomycetes species

**Figure 4:**
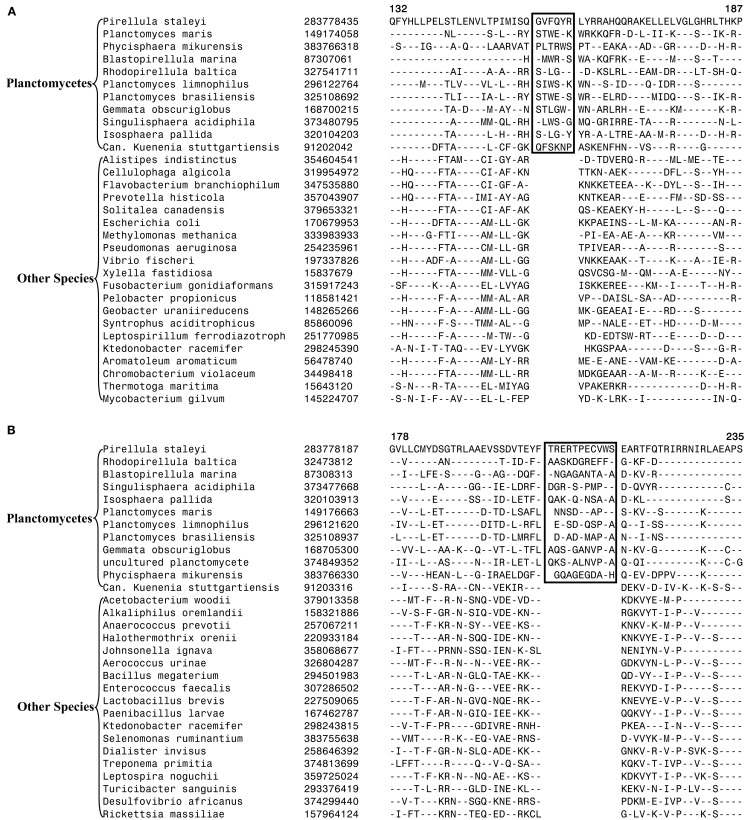
**Partial sequence alignments of (A) a conserved region within an ABC transporter protein depicting a 4 aa insert that is specifically present in in all sequenced Planctomycetes species; (B) An 11 aa insert in the cobyrinic acid ac-diamide synthase that is specific for all sequenced Planctomycetes except *Candidatus Kuenenia stuttgartiensis***.

**Figure A3:**
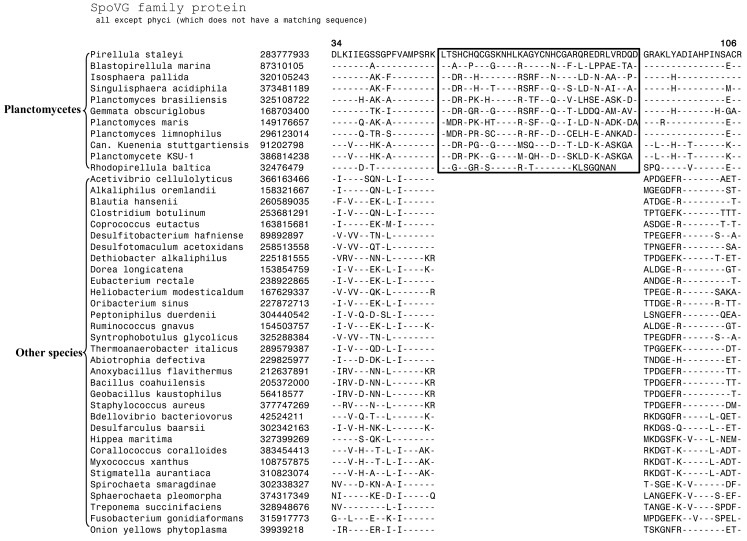
**A large, 32–36 aa insert present in all detected species of the Planctomycetes species is presented**. The conserved region is present within a conserved region of the SpoVG family protein and is not found in any organism outside of the Planctomycetes phylum.

The 11 Planctomycetes species for which sequences are available also formed a well-supported clade in our phylogenetic tree (Figure 1). The Planctomycetes species have been divided into two separate classes: the Phycisphaerae and the Planctomycetia (NCBI Taxonomy, 2012). *Phycisphaera mikurensis* is the sole recognized and sequenced species for the class Phycisphaerae. The Planctomycetia class is further divided into the orders Planctomycetales and Candidatus Brocadiales (Ward, 2011). The Candidatus Brocadiales consists of several candidate species including *K. stuttgartiensis*. Complete genomes for nine organisms from the order Planctomycetales are available: *Blastopirellula marina*, *Gemmata obscuriglobus*, *Isosphaera pallida*, *P. staleyi*, *Planctomyces* (*Pl*.) *brasiliensis*, *Pl. limnophilus*, *Pl. maris*, *Rhodopirellula baltica* and *Singulisphaera acidiphila*. The nine species of the Planctomycetales order, as expected, branched together in the tree. However, in conflict with the established placement of *K. stuttgartiensis* within the class Planctomycetia, this species was observed as the deepest branching member of the phylum with *Ph. mikurensis* sharing a closer relationship to the species of the Planctomycetales order. The deeper branching of the anammox species (viz. *K. stuttgartiensis*) in comparison to Phycisphaera has also been observed in earlier studies (Fukunaga et al., 2009; Fuchsman et al., 2012). Similar to the Verrucomicrobiae, no molecular or biochemical marker is known that is specific for the Planctomycetes species. However, two of the CSIs identified in this work were specific for all of the sequenced species from this phylum. The sequence information for one of these CSIs, consisting of a 6 aa insert in a conserved region of an ABC transporter protein is shown in Figure 4A. This CSI is uniquely present in all of the sequenced Planctomycetes species, but it is not found in any other bacteria. Similarly, in the SpoVG protein, which is involved in methicillin and glycopeptide resistance and production of extracellular polysaccharides in virulent *Staphylococcus aureus* (Matsuno and Sonenshein, 1999; Schulthess et al., 2009), a 36 aa insert in a conserved region is present in all of the sequenced Planctomycetes species (Figure A3 in Appendix). In view of the observed specificities of these CSIs for the species from the phylum Planctomycetes, they provide molecular markers for this phylum.

Another CSI identified in the present work supports the view that *K. stuttgartiensi*s represents a deep-branching group of organisms within the phylum Planctomycetes. In this case, a 10–11 aa insert in a conserved region of the protein cobyrinic acid ac-diamide synthase is present in all of the sequenced Planctomycetes species except *K. stuttgartiensis* (Figure 4B). The simplest and most likely explanation for the species distribution pattern of this CSI is that the genetic change leading to this insert was introduced into a common ancestor of other sequenced Planctomycetes species after the divergence of *K. stuttgartiensis*. Hence, the absence of this CSI from *K. stuttgartiensis* supports its position as the deepest branching sequenced species from this phylum, which is in agreement with its branching position in the phylogenetic trees (Figure 1; Fuchsman et al., 2012).

### Molecular markers for the larger clades within the PVC phyla of bacteria

Although the species of the phyla Planctomycetes, Verrucomicrobia, Lentisphaerae, and Chlamydiae formed distinct clades and branched in the proximity of each other in the phylogenetic tree based upon concatenated protein sequences (Figure 1), the grouping of these phyla into a single clade or other multi-phyla clades was very poorly supported by ML analysis, highlighting the concerns from earlier studies regarding amalgamation of these phyla into a single “superphylum” (Cho et al., 2004; Wagner and Horn, 2006; Griffiths and Gupta, 2007). Hence, molecular markers that could provide independent support for the grouping of these phyla are of much importance. Our analysis has identified a few molecular markers that are helpful in these regards.

**Figure 5:**
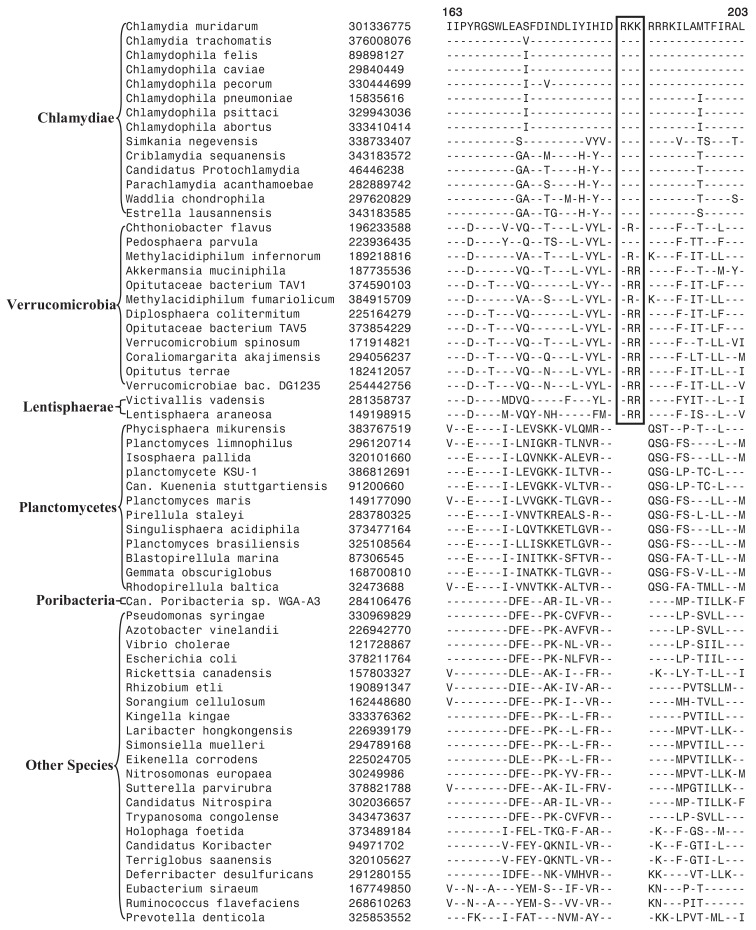
**A 3 aa insert in a conserved region of the RNA Polymerase β subunit (RpoB) that is specifically present in all sequenced Chlamydiae, Verrucomicrobia, and Lentisphaera species, but not found in Planctomycetes or any other phyla of bacteria**.

In our earlier work on Chlamydiae, a 3 aa insert in the β subunit of RNA polymerase (RpoB) was identified that in addition to the sequenced Chlamydiae species was also exclusively present in one Verrucomicrobia species (*V. spinosum*) whose sequence was available at that time (Griffiths and Gupta, 2007). An updating of the sequence information for this CSI (Figure 5) indicates that this CSI is specifically present in all members of the Chlamydiae and Verrucomicrobia phylum along with the two species of the phylum Lentisphaerae for which sequences are available. However, this CSI is not present in any other bacteria including different Planctomycetes and the Poribacteria. The unique shared presence of this conserved insert in this essential protein by all sequenced Chlamydiae, Verrucomicrobia, and Lentisphaerae species strongly indicates that the species from these three phyla shared a common ancestor exclusive of all other bacteria. Thus, the species distribution pattern of this CSI strongly supports the grouping together of these three phyla into a single large clade, consistent with their branching in the phylogenetic tree. The absence of this CSI in the Planctomycetes species is also consistent with its deeper branching in comparison to the other three phyla (Figure 1; Ward et al., 2000; Jenkins and Fuerst, 2001; Wagner and Horn, 2006; Griffiths and Gupta, 2007; Hou et al., 2008; Pilhofer et al., 2008).

**Figure 6:**
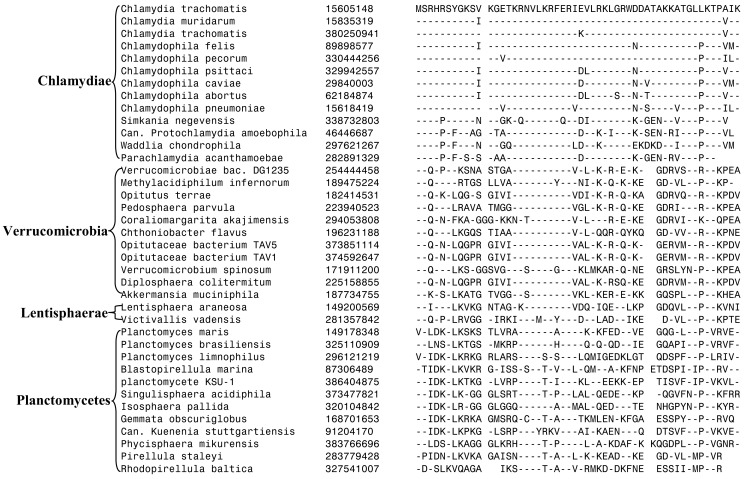
**Sequence alignment of a protein of unknown function that is uniquely found in various species from the PVC phylum of bacteria except Poribacteria**. In Blastp searches, no homolog of this protein is detected in any other bacteria outside of the PVC clade of bacteria.

Our detailed analysis identified no CSI that was specifically shared by all or most of species from the PVC phyla of bacteria. However, we have identified one signature protein, whose specific presence in various species belonging to the PVC clade suggests that the species from the four main phyla might be specifically related. The protein of interest is a hypothetical protein (the protein CT421.2 from *C. trachomatis*; accession number NP_219933) whose length varies from ∼53 aa in the Chlamydiaceae to more than 80 aa in the Planctomycetes. In BlastP searches with the *C. trachomatis* homolog all of the observed hits for this protein are for the PVC group of species and no hit outside of this group is observed. The 53 aa long region of this chlamydial protein is well conserved in all sequenced species belonging to the PVC clade and a sequence alignment for this region is presented in Figure 6. The specific presence of this protein in the PVC group of bacteria (all except Poribacteria) suggests that the gene for this protein initially originated in a common ancestor of these organisms, followed by its vertical transmission to various descendants. Although the function of this protein is not known, its specific presence in the PVC group of bacteria provides suggestive evidence that the species from these groups shared a common ancestor exclusive of other bacteria.

## Discussion and Conclusion

**Figure 7:**
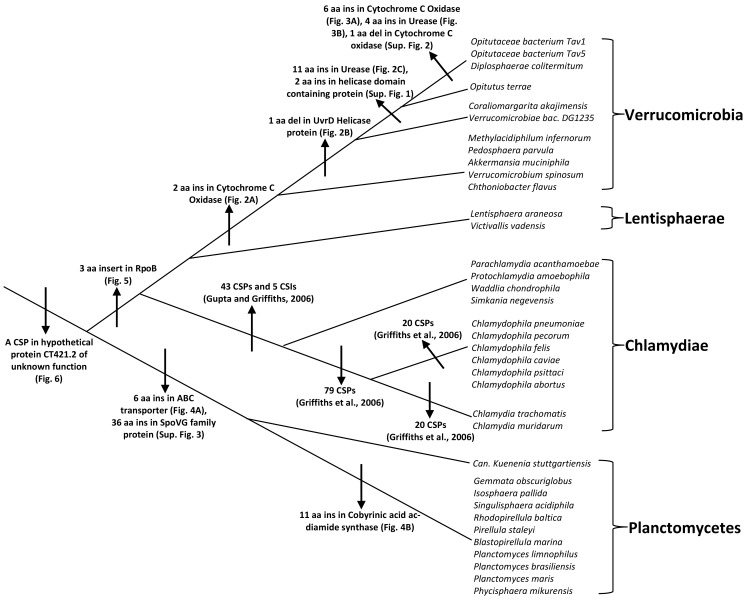
**A summary diagram depicting the different CSIs and CSPs that have been identified for the PVC clade of bacteria and the predicted evolutionary stages where the genetic changes leading for these molecular signatures likely originated**. Information for various CSIs and CSPs for the Chlamydiae is from our earlier work (Griffiths et al., 2005; Gupta and Griffiths, 2006; Griffiths and Gupta, 2007).

The PVC superphylum is proposed to be composed of numerous species that are part of four phyla and three candidate phyla. With several cellular features unique to members of this group of bacteria as well as the important pathogenic organisms present within this group, the relationships that these bacteria share with other prokaryotes and with each other is of great evolutionary interest (Devol, 2003; Sachse et al., 2009; Fuerst and Sagulenko, 2011; McInerney et al., 2011). However, elucidation of the relationships among the PVC group of bacteria has thus far proven difficult and led to contradictory results by phylogenetic means. In this work, we report for the first time identification of molecular markers in the form of CSIs and CSPs that are unique and distinctive characteristics of species from the phyla Verrucomicrobia and Planctomycetes and others that provide independent support for the grouping of species from the phyla Planctomycetes, Verrucomicrobia, Chlamydiae, and Lentisphaerae into larger clades. Large numbers of CSIs and CSPs for the Chlamydiae species were identified in our earlier work (Griffiths et al., 2005, 2006; Gupta and Griffiths, 2006). Based upon the species distribution patterns of these markers, the evolutionary stages where the genetic changes responsible for them have likely occurred are depicted in Figure 7.

Grounded upon the identified markers, it is now possible to clearly distinguish species from each of the three main phyla (viz. Planctomycetes, Verrucomicrobia, and Chlamydiae) that comprise the PVC clade of bacteria in molecular terms. The specificities of these markers for the species from these clades provide independent evidence for the monophyly of these clades. Additionally, based upon these molecular markers a number of relationships within these bacterial phyla can also be consolidated. Within Verrucomicrobia, newly identified CSIs allow the species from the class Opitutae and family Opitutaceae to be distinguished in molecular terms. The species distribution of these CSIs strongly indicate that the species *V. bacterium DG1235*, which is currently a part of the class Verrucomicrobiae, should in fact be transferred to the class Opitutae. A number of CSIs also provide evidence that the two unclassified species belonging to the family Opitutaceae viz. *O. bacterium TAV5* and *TAV1* are closely related to *D. colitermitum* and they should perhaps be assigned to the genus *Diplosphaera*. Within Planctomycetes, the species distribution pattern of the identified CSIs strongly indicates that the anammox species *K. stuttgartiensis* constitutes the deepest branching lineage of this phylum, which is consistent with its branching in the phylogenetic tree. However, this inference is at variance with the current assignment of *K. stuttgartiensis* to the class Planctomycetia, whereas the species *Ph. mikurensis* which branches less deeply than *K. stuttgartiensis* is part of a separate class (Phycisphaerae). The anammox organisms such as *K. stuttgartiensis* possess a number of distinctive features such as the presence of an ammonium oxidizing organelle called the anammoxosome and cell division by constrictive binary fission, which differentiate them from other members of the class Planctomycetia (van Niftrik et al., 2009).

More importantly, in the present work, we have also identified some signatures that are helpful in clarifying how the species from the PVC phyla of bacteria are related and providing some evidence supporting their amalgamation into larger clades. However, only a couple of signatures that are helpful in this regard were identified. The most significant of these signatures is a 3 aa long insert in the RpoB protein that is commonly and uniquely shared by all of the sequenced Chlamydiae, Verrucomicrobia, and Lentisphaerae species but not found in any other bacteria. The observed species specificity of this signature, in this important protein, strongly indicates that the species from these three phyla shared a common ancestor exclusive of all other bacteria. The RpoB protein also contains a number of other CSIs in other regions of the protein that are specific for other groups/phyla of bacteria (Griffiths and Gupta, 2007; Gupta and Mok, 2007; Gao et al., 2009; Gupta and Bhandari, 2011). The high degree of specificity of these CSIs for different groups/phyla of bacteria provides evidence that the gene for RpoB has not been laterally transferred among different bacterial groups. An other signature that is informative in this regard consists of a small protein of unknown function that is specifically found in all of the species from the above three phyla of bacteria and also in the Planctomycetes. The observed species specificity of this protein suggests that the gene for this protein very likely originated in a common ancestor of the PVC clade of bacteria. However, in this case other possibilities to account for the species distribution of this protein cannot be entirely excluded. Nonetheless, the unique shared presence of this protein by various species that are part of the PVC clade provide evidence supporting their grouping into a large clade.

The molecular markers described in the present work, in addition to their usefulness for evolutionary and taxonomic studies, also provide novel and valuable tools for the identification of these organisms in different environments. In view of the presence of the identified CSIs in conserved regions of various proteins, degenerate primers based upon conserved regions in them can be designed for selective amplification (detection) of sequences from various species from these groups. Additionally, blast searches with the sequence queries based upon these proteins also provide useful identification tools for detection of both known and unknown species from these phyla in metagenomic sequences. Finally, the identified CSIs and CSP provide novel tools for genetic and biochemical studies and functional studies on them could lead to discovery of novel biochemical and/or physiochemical properties that are commonly shared by these phyla or the PVC clade of bacteria.

## Conflict of Interest Statement

The authors declare that the research was conducted in the absence of any commercial or financial relationships that could be construed as a potential conflict of interest.
